# Wearable biosensors characterize Baguazhang exercise intensity, trunk acceleration, and cardiovascular autonomic adaptations in postmenopausal women

**DOI:** 10.3389/fphys.2026.1922459

**Published:** 2026-09-18

**Authors:** Jinjin Cao, Meijuan Zhang

**Affiliations:** 1 School of Physical Education, Xuzhou University of Technology, Xuzhou, China; 2 Department of Physical Education, Beijing Union University, Beijing, China

**Keywords:** accelerometry, Baguazhang, cardiovascular autonomic function, exercise physiology, heart rate variability, postmenopausal women, training load, wearable biosensors

## Abstract

**Background:**

Baguazhang is a traditional Chinese mind-body exercise that combines circular locomotion, repeated changes of direction, lower-limb postural control, trunk rotation, coordinated upper-limb movements, and breathing regulation. These features make it a multicomponent movement practice, but its cardiovascular intensity and trunk-acceleration profile have not been well quantified in postmenopausal women.

**Methods:**

Forty-six sedentary postmenopausal women were randomly assigned to a Baguazhang intervention group or a habitual-lifestyle control group in a parallel-group randomized controlled trial. The intervention group completed 12 weeks of supervised Baguazhang training (three 60-min sessions/week). Synchronized chest-strap electrocardiography and waist-mounted tri-axial accelerometry quantified heart-rate-derived internal load and waist-derived acceleration amplitude. The primary outcome was systolic blood pressure (SBP). Secondary outcomes were diastolic blood pressure (DBP), resting heart rate, body composition, and LnRMSSD. Phase-specific sensor data were summarized for the warm-up, mud-wading, circle-walking, and cool-down blocks. Between-group outcome effects were estimated with baseline-adjusted ANCOVA.

**Results:**

Across 812 attended sessions with valid recordings, mean session heart rate was 118.5 ± 8.4 beats/min (45.7 ± 6.2% HRR) and mean session TRIMP was 59.7 ± 9.3 arbitrary units. The circle-walking block showed the highest phase-specific cardiovascular demand (133.4 ± 9.2 beats/min; 61.5 ± 7.4% HRR) and the largest phase TRIMP (37.5 ± 5.6 AU); waist-derived MAD during this block was 0.22 ± 0.05 g. For the primary outcome, SBP was lower after Baguazhang than after habitual lifestyle (adjusted mean difference −7.2 mmHg, 95% CI −10.5 to −3.9; p < 0.001). Supportive secondary analyses, without multiplicity adjustment, yielded adjusted differences in DBP (−4.4 mmHg), resting heart rate (−4.5 beats/min), and LnRMSSD (+0.31), with smaller nominal differences in body mass index and body-fat percentage.

**Conclusion:**

Baguazhang elicited moderate average cardiovascular intensity, with the greatest phase-specific demand during the circle-walking block. After 12 weeks, SBP was lower than in habitual-lifestyle controls. Secondary outcomes should be interpreted as supportive. Waist accelerometry characterized trunk-acceleration amplitude but did not measure joint or musculoskeletal loading. The findings support Baguazhang as a feasible, measurable, moderate-intensity multicomponent exercise option but do not establish superiority over other exercise modalities.

## 1 Introduction

The menopausal transition is accompanied by a decline in endogenous estrogen, unfavorable changes in vascular function and body composition, and a shift toward sympathetic predominance. These changes contribute to higher cardiovascular risk, elevated blood pressure, metabolic vulnerability, and reduced autonomic flexibility in postmenopausal women (Rosano et al., 2007; Thayer et al., 2010; Valensi, 2021; Yang et al., 2024). Because these risks are strongly influenced by behavior, non-pharmacological interventions that are safe, acceptable, and sustainable are central to healthy aging in this population.

Regular physical activity is one of the most effective strategies for maintaining cardiovascular and metabolic health across adulthood (World Health Organization, 2020). Nevertheless, many postmenopausal women do not meet recommended levels of moderate-to-vigorous physical activity. Barriers include musculoskeletal discomfort, fear of injury, limited exercise confidence, and low motivation for conventional aerobic training. Exercise modalities that combine an adequate cardiovascular stimulus with coordinated movement, postural control, and an accessible learning structure may therefore be relevant to this population.

Traditional Chinese martial arts are increasingly studied as mind-body exercise modalities that integrate dynamic movement, postural control, attentional focus, and breathing regulation. Tai Chi and Baduanjin have been examined more widely than other forms and have shown potential benefits for balance, sleep quality, musculoskeletal function, and cardiometabolic outcomes (Li et al., 2012; Zou et al., 2018). Baguazhang, also known as Eight Trigram Palm, remains less studied despite a distinctive movement structure that combines circle walking, repeated directional changes, a relatively lowered center-of-mass posture, mud-wading steps, trunk rotation, coordinated palm changes, and breathing regulation. At the movement-pattern level, this combination differs from steady straight-line walking and is best considered a multicomponent practice. Whether these structural features confer benefits beyond those of other forms of moderate-intensity exercise was not tested in the present study.

A major barrier to the physiological interpretation of Baguazhang is the lack of objective exercise-dose data. Laboratory metabolic testing can quantify oxygen consumption but may disrupt natural rotational movement and circular walking. Subjective measures such as ratings of perceived exertion are useful but may underestimate physiological strain during attentional or meditative movement practices (Borg, 1982). Step counts alone are also insufficient because Baguazhang includes postural stabilization and rotational control that may increase internal cardiovascular demand without a proportional increase in external movement amplitude.

Wearable biosensors provide a practical approach for quantifying exercise dose in ecologically valid settings. Chest-strap electrocardiographic sensors can provide high-resolution R-R interval and heart-rate data, while inertial sensors can quantify movement acceleration through tri-axial accelerometry (Seshadri et al., 2019; Majumder et al., 2017). Compared with wrist-based photoplethysmography, which can be affected by motion artifacts during vigorous arm movement, chest-strap and electrocardiography-based measurements are better suited for heart-rate variability analysis during dynamic exercise (Gilgen-Ammann et al., 2019; Bent et al., 2020; Schaffarczyk et al., 2022). Combining physiological and kinematic signals can therefore characterize cardiovascular demand and concurrent trunk-acceleration patterns without equating waist accelerometry with ground-reaction force or joint loading.

The present study aimed to characterize the internal cardiovascular load and waist-derived trunk-acceleration profile of Baguazhang using synchronized wearable biosensors and to evaluate the effects of a 12-week supervised Baguazhang intervention on cardiovascular and autonomic outcomes in sedentary postmenopausal women. The primary outcome was systolic blood pressure (SBP). Secondary outcomes were diastolic blood pressure (DBP), resting heart rate, heart rate variability (LnRMSSD), and body composition. The study compared Baguazhang with habitual lifestyle and was not designed to test superiority over walking or another active exercise program.

## 2 Materials and methods

### 2.1 Study design, participants, and ethics

This study was designed as a parallel-group randomized controlled trial with blinded outcome assessment. The study followed the principles of the Declaration of Helsinki (World Medical Association, 2025) and was approved by the Institutional Review Board of Xuzhou University of Technology (protocol code 2025-BGZ-08; approval date: 15 January 2025). All participants provided written informed consent before enrollment.

Trial reporting followed the Consolidated Standards of Reporting Trials (CONSORT) 2025 recommendations (Hopewell et al., 2025), and participant flow is presented in **Figure 1**. This study was not prospectively registered in a publicly accessible clinical trial registry. However, to ensure methodological integrity and that the outcome hierarchy was determined before data collection, the complete study protocol was prospectively reviewed and approved by the Institutional Review Board (IRB approval code: 2025-BGZ-08; approval date: 15 January 2025) prior to participant enrollment. In accordance with this rigorously approved *a priori* design, SBP was strictly designated as the single primary outcome and formed the basis for the pre-trial sample-size calculation. DBP, resting heart rate, LnRMSSD, body mass index, and body-fat percentage were predefined as secondary outcomes, while participant-level sensor-outcome correlation analyses were exploratory.

**Figure 1:**
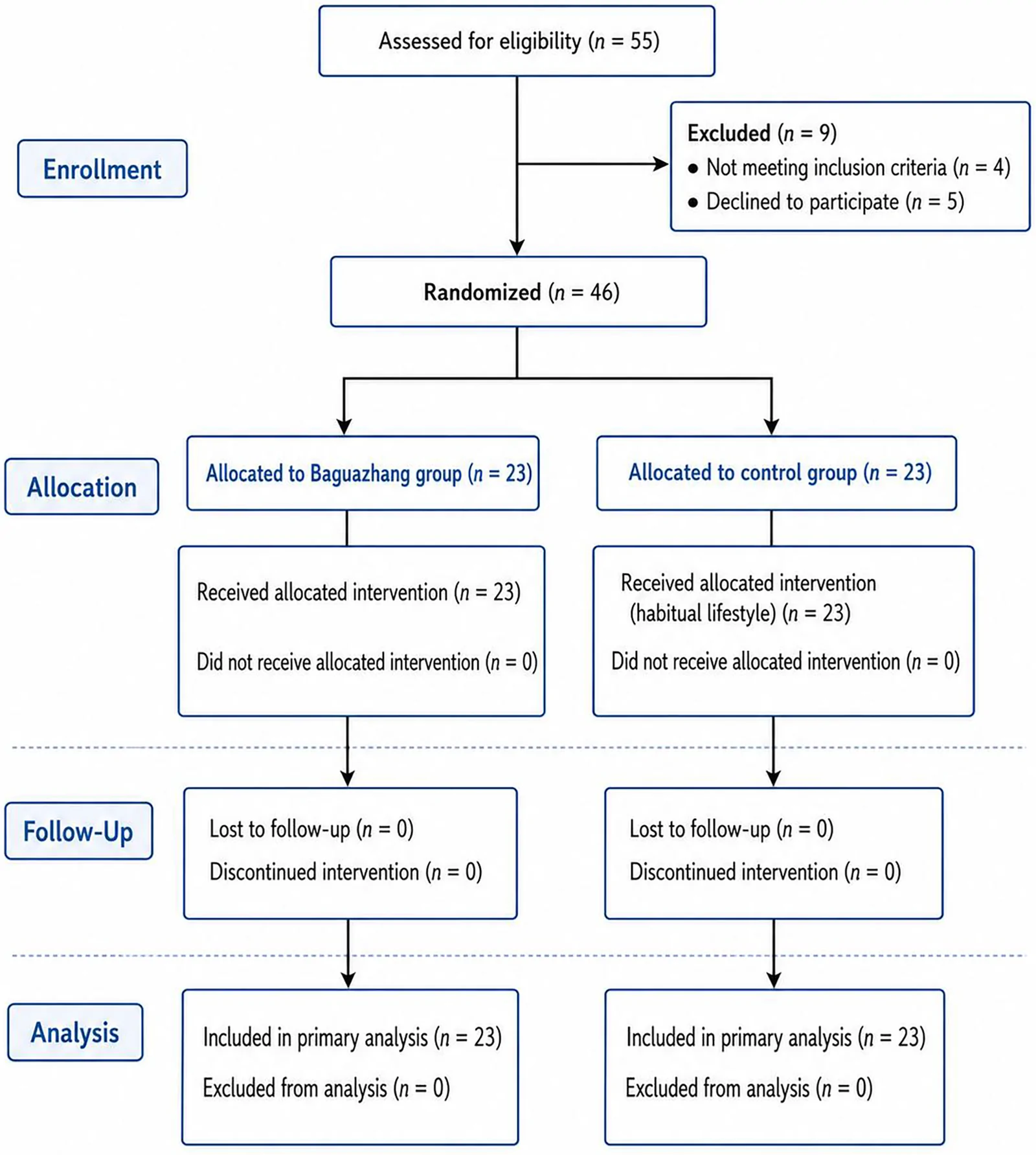
CONSORT flow diagram of participant screening, enrollment, randomization, follow-up, and analysis.

Participants were recruited from local communities through digital advertisements and community health talks between February 2025 and March 2025. Eligibility criteria were biological female sex, age 50–65 years, natural menopause defined as amenorrhea for at least 12 consecutive months, sedentary status at baseline (<150 min/week of moderate-to-vigorous physical activity), and no previous martial-arts training. Exclusion criteria were uncontrolled hypertension (>160/100 mmHg), a history of clinically significant cardiac arrhythmia, severe musculoskeletal injury limiting weight-bearing exercise, current beta-blocker use, or current hormone replacement therapy.

An *a priori* power analysis using G*Power 3.1 indicated that 40 participants (20 per group) would provide 80% power to detect a medium group-by-time effect (f = 0.25) in the primary outcome (systolic blood pressure) at an alpha level of 0.05. This effect size was estimated based on prior meta-analytical data regarding moderate-intensity mind-body exercise in postmenopausal cohorts (Yang et al., 2024), assuming a moderate correlation between repeated measures (r = 0.5) and applying a non-sphericity correction. To allow for attrition, 55 women were initially screened. Nine were excluded (four did not meet inclusion criteria, five declined to participate), resulting in forty-six eligible participants.

Participants were randomized (1:1 ratio) to the Baguazhang group (n = 23) or the control group (n = 23). The randomization allocation sequence was generated by an independent statistician using block randomization (block size of 4) via a computer random-number generator. Allocation concealment was maintained using sequentially numbered, opaque, sealed envelopes. The primary investigator enrolled the participants, and an independent research assistant opened the envelopes and assigned participants to interventions. To minimize bias, outcome assessors and data analysts were blinded to group allocation throughout the trial. Participants could not be blinded due to the nature of the active exercise intervention.

### 2.2 Wearable sensor system

The body sensor network consisted of a chest-strap electrocardiographic sensor and a waist-mounted tri-axial accelerometer. Heart-rate and device-generated R-R interval data, alongside raw electrocardiographic (ECG) signals (sampled at 130 Hz), were collected with the Polar H10 sensor (Polar Electro Oy, Kempele, Finland), which has been validated for heart-rate variability analysis during rest and incremental exercise (Schaffarczyk et al., 2022). Kinematic data were recorded with an ActiGraph wGT3X-BT accelerometer (ActiGraph LLC, Pensacola, FL, United States), positioned at the L3 lumbar level to approximate center-of-mass movement. The accelerometer was set to sample at 100 Hz with a dynamic range of ±8 g.

A custom Android application functioned as the mobile gateway for simultaneous sensor acquisition. Incoming data streams were timestamped and synchronized using a Network Time Protocol (NTP) server to correct for clock drift and Bluetooth transmission latency before offline analysis. Missing data packets were handled via last-observation-carried-forward for signal gaps under 1 second, whereas gaps exceeding 1 second were marked as missing and excluded from the specific epoch calculation. This configuration enabled concurrent assessment of heart-rate-derived internal load and accelerometry-derived movement amplitude. The experimental setup is shown in **Figure 2**.

**Figure 2:**
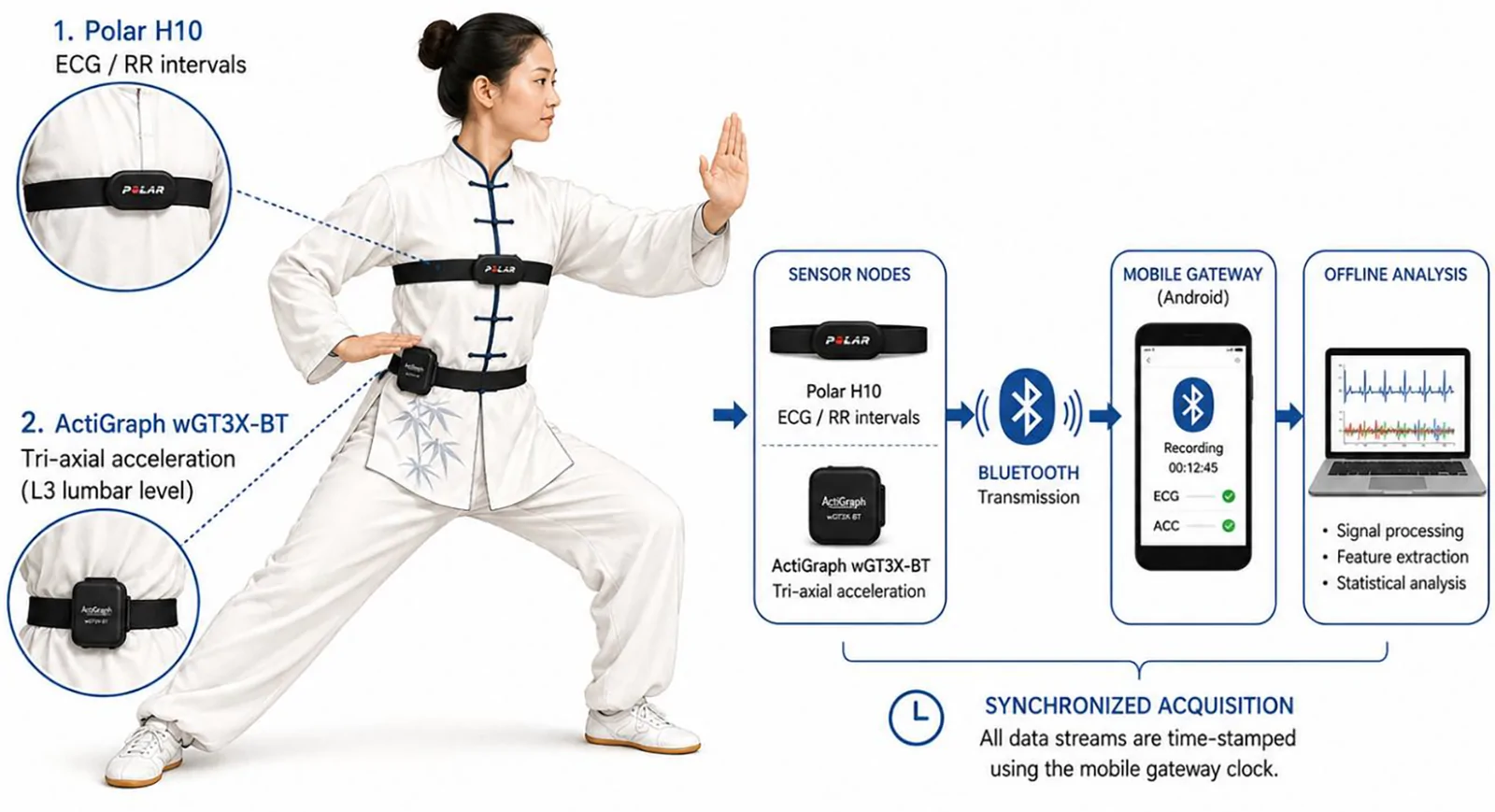
Wearable sensor architecture and experimental setup. The body sensor network combined chest-strap electrocardiographic monitoring and waist-mounted accelerometry, with synchronized transmission to a mobile gateway for offline analysis.

### 2.3 Signal processing and feature extraction

All sensor data were processed in MATLAB R2023a (MathWorks, Natick, MA, United States). The custom signal-processing scripts are available upon reasonable request.

Electrocardiographic signals were filtered with a fourth-order Butterworth band-pass filter from 0.5 to 30 Hz to reduce baseline wander and high-frequency noise. R-wave peaks were detected using the Pan-Tompkins algorithm (Pan and Tompkins, 1985). R-R interval series were screened for ectopic beats and motion artifacts; intervals deviating by more than 20% from the local median within a 10-beat window were replaced by cubic spline interpolation (Mateo and Laguna, 2003). Following automated correction, all ECG traces were visually inspected by a blinded data analyst to ensure artifact removal accuracy. The interpolation rate remained consistently below 3% of the total beats per session.

Internal load was quantified with Banister’s training impulse (TRIMP), which integrates exercise duration and relative heart-rate strain (Banister, 1991). Heart-rate reserve was calculated epoch by epoch as HRr(t) = [HR(t) − HRrest]/[HRmax − HRrest]. For each participant, HRrest was defined as the resting heart rate obtained during the standardized baseline physiological assessment and was held constant for all training-session HRR and TRIMP calculations throughout the 12-week intervention. The weekly pre-session resting heart-rate measurements presented descriptively in **Figure 3** were not used in the HRR or TRIMP calculations. Age-predicted maximal heart rate was estimated with the Tanaka equation, HRmax = 208 − 0.7 × age (Tanaka et al., 2001). TRIMP was calculated epoch by epoch as TRIMP = Σ[Δt × HRr(t) × 0.86 × exp(1.67 × HRr(t))], where Δt is the epoch duration in minutes, 0.86 is the female-specific multiplicative coefficient, and 1.67 is the female-specific exponential weighting factor.

**Figure 3:**
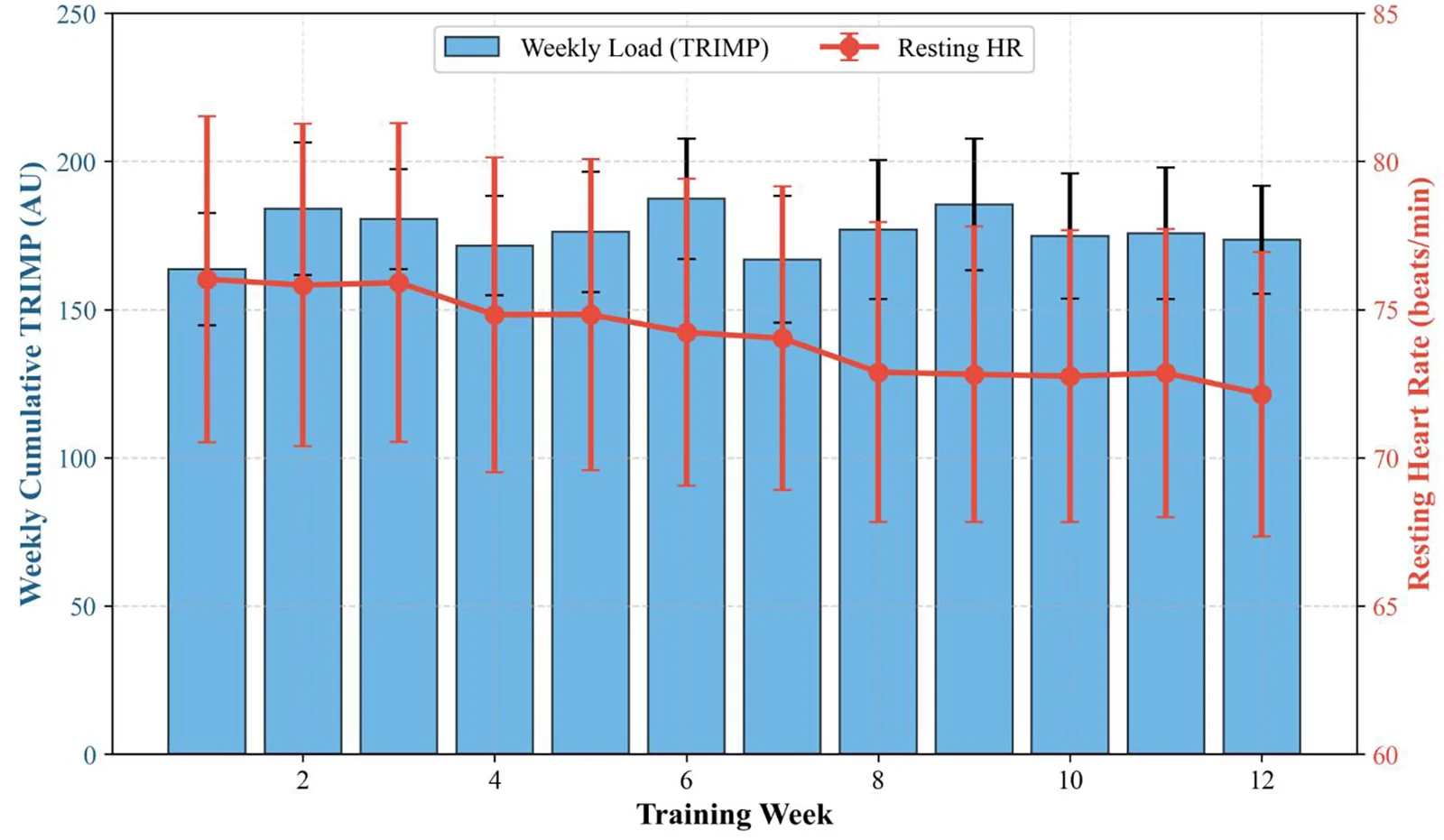
Weekly cumulative training impulse and weekly resting heart-rate trend during the 12-week intervention. Bars show mean weekly cumulative Banister training impulse (TRIMP) with error bars; the line and circular markers show mean pre-session resting heart rate recorded at the first supervised session of each week. Error bars indicate between-participant standard deviations. Weekly values are presented descriptively and were not used to quantify test-retest reproducibility or as independent efficacy endpoints.

Accelerometry-derived movement amplitude was quantified from tri-axial acceleration using vector magnitude (VM) and mean amplitude deviation (MAD). For each sample i, raw vector magnitude was calculated as VM_i = sqrt(x_i^2 + y_i^2 + z_i^2). The dynamic acceleration component was obtained by removing the 1-g gravitational component. MAD was then calculated within 5-s epochs as MAD = (1/N)Σ|VM_i − mean(VM)|, representing the dynamic variation in movement amplitude expressed in gravitational units (g). MAD has been validated as an accelerometer-based indicator of ambulatory movement intensity and is less dependent on sensor orientation than single-axis outputs (Vähä-Ypyä et al., 2015). MAD describes acceleration variability at the waist and is not a direct measure of ground-reaction force, joint loading, loading rate, or tissue mechanical stress. Step counts were estimated by applying a peak-detection algorithm to the vertical acceleration axis after a 3-Hz low-pass filter. The data-processing workflow is summarized in **Figure 4**.

**Figure 4:**
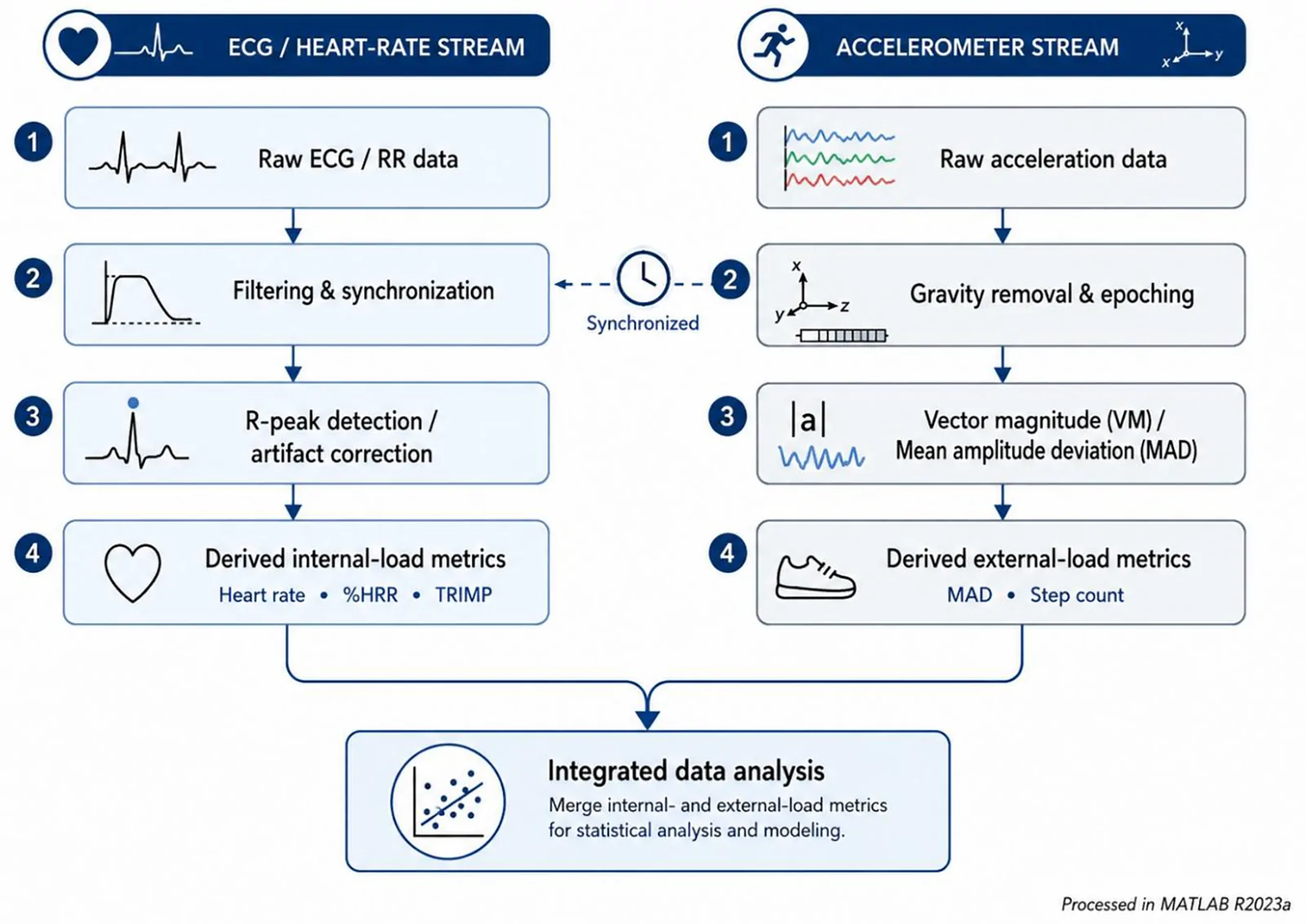
Signal-processing and feature-extraction workflow. Raw ECG/RR and tri-axial acceleration streams were synchronized, cleaned, and processed to derive internal-load metrics (heart rate, %HRR, and TRIMP) and accelerometry-derived movement metrics (MAD and step count) before integrated statistical analysis.

For phase-specific characterization, each valid 60-min training recording was segmented according to the standardized session schedule: warm-up (0–10 min), mud-wading drills (10–25 min), circle-walking block (25–50 min), and cool-down (50–60 min). During Weeks 1–4, the circle-walking block included planned active-recovery and technical-correction intervals as part of the familiarization strategy; from Week 5 onward, the 25-min block was performed continuously. Average heart rate, %HRR, phase TRIMP, MAD, and step count were calculated separately for each block. Within each participant, phase-specific values were first averaged across all attended sessions with valid recordings, and group summaries were then calculated from the resulting participant-level averages (n = 23). The 812 valid sessions therefore contributed repeated observations within participants and were not treated as independent statistical units.

### 2.4 Intervention protocol

Participants in the Baguazhang group completed a 12-week supervised training program consisting of three 60-min sessions per week. Sessions were led by a certified traditional martial arts coach with more than 10 years of teaching experience and followed a standardized Old Eight Palms curriculum. Each session retained four fixed time blocks: a 10-min warm-up (joint mobilization and dynamic stretching), 15 min of mud-wading drills, a 25-min circle-walking block, and a 10-min cool-down (static stretching and breathing regulation). Because participants had no previous martial-arts experience, technical familiarization was concentrated in the first 4 weeks. During Weeks 1–2, the 25-min circle-walking block was divided into five 5-min cycles, each comprising approximately 4 min of walking and 1 min of active recovery with technical correction. During Weeks 3–4, the block was organized as two extended walking intervals of approximately 10 min separated by active-recovery/technical-correction periods totaling 5 min. From Week 5 onward, participants performed the full 25-min circle-walking block continuously with integrated single-palm and double-palm changes. The week-by-week curriculum is presented in **Table 1**.

**Table 1:** Twelve-week supervised Baguazhang curriculum and technical familiarization.

Week	Technical focus/familiarization	Mud-wading drills (15 min)	Circle-walking block (25 min)	Warm-up + cool-down (20 min)	Cadence/intended intensity
1	Basic-posture familiarization and lower-limb endurance through interval practice	Linear mud-wading steps; emphasis on foot placement	5 × (≈4 min walking + 1 min active recovery/technical correction)	Joint mobilization/dynamic stretching; static stretching and natural breathing	70–80 steps/min during walking intervals; light-to-moderate response monitored
2	Basic-posture familiarization and lower-limb endurance through interval practice	Linear mud-wading steps; emphasis on foot placement	5 × (≈4 min walking + 1 min active recovery/technical correction)	Joint mobilization/dynamic stretching; static stretching and natural breathing	70–80 steps/min during walking intervals; light-to-moderate response monitored
3	Integration of upper-body postures and palm changes with continuous stepping	Circular mud-wading steps; emphasis on center-of-mass control	2 × (≈10 min walking + ≈2.5 min active recovery/technical correction); single- and double-palm changes introduced progressively	Joint mobilization/dynamic stretching; static stretching and breathing regulation	70–80 steps/min; intended moderate overall session response
4	Integration of upper-body postures and palm changes with continuous stepping	Circular mud-wading steps; emphasis on center-of-mass control	2 × (≈10 min walking + ≈2.5 min active recovery/technical correction); single- and double-palm changes integrated	Joint mobilization/dynamic stretching; static stretching and breathing regulation	70–80 steps/min; intended moderate overall session response
5	Maintain technical stability during prolonged continuous practice	Circular mud-wading steps with coordinated trunk rotation	25 min uninterrupted circle walking with single- and double-palm changes	Joint mobilization/dynamic stretching; static stretching and breathing regulation	70–80 steps/min; standardized moderate session target; HR/HRR reviewed
6	Maintain technical stability during prolonged continuous practice	Circular mud-wading steps with coordinated trunk rotation	25 min uninterrupted circle walking with single- and double-palm changes	Joint mobilization/dynamic stretching; static stretching and breathing regulation	70–80 steps/min; standardized moderate session target; HR/HRR reviewed
7	Maintain technical stability during prolonged continuous practice	Circular mud-wading steps with coordinated trunk rotation	25 min uninterrupted circle walking with single- and double-palm changes	Joint mobilization/dynamic stretching; static stretching and breathing regulation	70–80 steps/min; standardized moderate session target; HR/HRR reviewed
8	Maintain technical stability during prolonged continuous practice	Circular mud-wading steps with coordinated trunk rotation	25 min uninterrupted circle walking with single- and double-palm changes	Joint mobilization/dynamic stretching; static stretching and breathing regulation	70–80 steps/min; standardized moderate session target; HR/HRR reviewed
9	Maintain technical stability during prolonged continuous practice	Circular mud-wading steps with coordinated trunk rotation	25 min uninterrupted circle walking with single- and double-palm changes	Joint mobilization/dynamic stretching; static stretching and breathing regulation	70–80 steps/min; standardized moderate session target; HR/HRR reviewed
10	Maintain technical stability during prolonged continuous practice	Circular mud-wading steps with coordinated trunk rotation	25 min uninterrupted circle walking with single- and double-palm changes	Joint mobilization/dynamic stretching; static stretching and breathing regulation	70–80 steps/min; standardized moderate session target; HR/HRR reviewed
11	Maintain technical stability during prolonged continuous practice	Circular mud-wading steps with coordinated trunk rotation	25 min uninterrupted circle walking with single- and double-palm changes	Joint mobilization/dynamic stretching; static stretching and breathing regulation	70–80 steps/min; standardized moderate session target; HR/HRR reviewed
12	Maintain technical stability during prolonged continuous practice	Circular mud-wading steps with coordinated trunk rotation	25 min uninterrupted circle walking with single- and double-palm changes	Joint mobilization/dynamic stretching; static stretching and breathing regulation	70–80 steps/min; standardized moderate session target; HR/HRR reviewed

Technical progression emphasized movement competence, coordinated palm changes, postural stability, and the duration of uninterrupted practice rather than progressive aerobic overload. Walking cadence was standardized at approximately 70–80 steps/min and regulated by the instructor’s verbal cues. Heart rate and HRR were reviewed after each session to verify that the intended overall session demand remained within a moderate range. The intervention therefore combined mobility/flexibility, locomotor endurance, lower-limb postural demands, dynamic balance, trunk–limb coordination, and breathing regulation; the present study directly quantified cardiovascular load and waist-derived acceleration, but did not directly measure balance performance, muscular endurance, or motor-learning outcomes.

Adherence was continuously monitored, and a minimum attendance rate of 80% (29 out of 36 sessions) was targeted. All adverse events, including falls, musculoskeletal pain, dizziness, and exercise intolerance, were systematically monitored and recorded during every session.

Participants in the control group were instructed to maintain their habitual lifestyle and not to begin any new structured exercise program or alter their dietary habits during the study period. Both groups maintained daily activity logs, which were reviewed bi-weekly by the research team to monitor step counts (via participants’ personal smartphones) and detect any significant changes in physical activity, sleep, or stress levels.

### 2.5 Outcome measures

Assessments were conducted at baseline and after the 12-week intervention in a temperature-controlled laboratory (maintained at 22–24 °C and 45–55% relative humidity). To minimize the influence of circadian variations, all physiological tests were conducted in the morning (08:00–10:00 AM). Participants were instructed to avoid caffeine, alcohol, smoking, vigorous exercise, and medication changes for 24 hours before testing, and to ensure at least 7 hours of sleep.

The primary outcome, SBP, and the secondary outcome, DBP, were measured using an automated oscillometric device (Omron HEM-7130, Omron Healthcare, Kyoto, Japan; validated according to international protocols). Measurements were taken on the non-dominant arm using an appropriately sized cuff. After 15 min of supine rest, three consecutive blood pressure readings were recorded with 1-min intervals. The average of the three readings was used for analysis.

Body composition, including body mass index and body fat percentage, was assessed by bioelectrical impedance analysis (InBody 770, InBody Co., Seoul, South Korea). To standardize hydration and digestion, participants were tested following an overnight fast (at least 8 hours without food or water) and after emptying their bladders.

Autonomic function was assessed using a 5-min resting R-R interval recording in the supine position, following accepted standards for heart rate variability measurement and interpretation (Task Force, 1996; Billman, 2011). Resting HRV data were captured using the Polar H10 chest strap immediately following the resting blood pressure measurement. Breathing was not mechanically paced to avoid acute stress, but participants were instructed to maintain a relaxed, spontaneous breathing rhythm.

Data were analyzed using Kubios HRV Standard software (version 3.5, Kubios Oy, Kuopio, Finland). Time-domain measures included the root mean square of successive differences (RMSSD). Because RMSSD data are typically skewed, values were log-transformed (LnRMSSD) prior to statistical analysis. LnRMSSD was analyzed as the principal autonomic secondary outcome, reflecting parasympathetic modulation. Additionally, to generate the weekly resting heart-rate trend over the 12 weeks, pre-session resting heart rate was recorded for 5 minutes at the beginning of the first training session of each week for the intervention group.

### 2.6 Statistical analysis

All statistical analyses followed the intention-to-treat (ITT) principle, with all 46 randomized participants analyzed according to their originally assigned groups. All participants completed both baseline and post-intervention assessments, and complete data were available for the primary and secondary clinical outcomes; therefore, no endpoint-data imputation was required. Missing intervention-session observations were treated separately: of the 828 planned Baguazhang sessions, 16 were not attended because of personal scheduling conflicts and consequently generated no session data. These missed sessions were not imputed. Brief sensor-packet gaps within recorded sessions were handled at the signal-processing stage as described in Section 2.2 and were distinct from missing clinical outcomes or missed sessions. Data are presented as mean ± standard deviation unless otherwise indicated. Normality of continuous variables was assessed using the Shapiro-Wilk test, and homogeneity of variances was assessed using Levene’s test. Baseline characteristics were summarized descriptively rather than evaluated with significance tests.

Intervention effects were evaluated using analysis of covariance (ANCOVA). Post-intervention values were used as the dependent variables, group assignment as the fixed factor, and the respective baseline values as covariates. Adjusted mean differences between groups are reported with 95% confidence intervals (CIs), exact p values, and partial eta squared (η²p) effect sizes.

Pearson correlation coefficients were used to explore associations between sensor-derived training measures and changes in health outcomes within the Baguazhang group. Mean session TRIMP was used to characterize the typical internal load of an attended session, whereas cumulative 12-week TRIMP was used as the participant-level exposure variable in exploratory correlation analyses. For each participant, cumulative TRIMP was calculated as the arithmetic sum of session-specific TRIMP values across all attended sessions with valid sensor recordings. All 812 attended sessions yielded valid recordings and were included; the 16 missed sessions were not assigned estimated or imputed TRIMP values. Mean MAD was calculated as a participant-level average across valid attended sessions. Correlation analyses therefore used one value per participant (n = 23), avoiding session-level pseudoreplication. These analyses were exploratory and were not adjusted for multiple comparisons. Phase-specific sensor metrics were summarized descriptively from participant-level averages, and no inferential comparisons among session phases were performed.

Analyses were conducted using SPSS 27.0 (IBM Corp., Armonk, NY, United States). SBP was the single primary outcome and was evaluated with a two-sided alpha level of 0.05 without multiplicity adjustment. DBP, LnRMSSD, resting heart rate, body mass index, and body-fat percentage were secondary supportive outcomes. No multiplicity adjustment was applied to these secondary endpoints; their p values are nominal and should be interpreted as supportive rather than confirmatory.

Furthermore, to account for baseline variance and potential regression to the mean, a parsimonious multivariable linear regression analysis was conducted with post-intervention SBP as the dependent variable, and total accumulated TRIMP and baseline SBP as predictors. Unstandardized regression coefficients (B), 95% CIs, and adjusted R² were reported.

## 3 Results

### 3.1 Participant characteristics and sensor data quality

Baseline characteristics are summarized descriptively in **Table 2**. The two randomized groups showed broadly similar distributions of age, anthropometric measures, years since menopause, systolic blood pressure, and resting heart rate. Baseline values were incorporated as covariates in the ANCOVA models used for intervention-effect estimation. The cohort showed characteristics commonly observed in sedentary postmenopausal women, including body mass index values near the overweight range and slightly elevated systolic blood pressure.

**Table 2:** Baseline characteristics of participants.

Variable	Baguazhang group (n = 23)	Control group (n = 23)
Age (years)	56.4 ± 4.2	57.1 ± 3.8
Height (cm)	161.2 ± 5.4	160.8 ± 4.9
Weight (kg)	64.5 ± 8.1	65.2 ± 7.6
Body mass index (kg/m²)	24.8 ± 3.1	25.1 ± 3.4
Years since menopause	5.2 ± 3.4	5.8 ± 3.1
Systolic blood pressure (mmHg)	134.5 ± 9.2	133.8 ± 8.9
Resting heart rate (beats/min)	76.4 ± 5.5	75.8 ± 6.1

Values are mean ± SD. Baseline characteristics were summarized descriptively; no between-group significance tests were used to assess baseline balance.

All 46 randomized participants (23 per group) completed both baseline and post-intervention assessments and were included in the intention-to-treat analysis. No participants were lost to follow-up, withdrew, or were excluded, and no primary or secondary endpoint data required imputation.

Across the supervised intervention period, 828 training sessions were planned (23 participants × 36 sessions). Of these, 812 sessions were attended and recorded with valid wear time, corresponding to an adherence rate of 98.1%. The 16 missed sessions were due to minor personal scheduling conflicts. No adverse events, falls, or exercise-intolerance episodes were reported. The wearable sensor system demonstrated stable acquisition, with a data packet loss rate of less than 2.5%. **Figure 5** shows one representative session for signal visualization only; group-level phase-specific cardiovascular and acceleration summaries are reported separately in **Table 3**.

**Figure 5:**
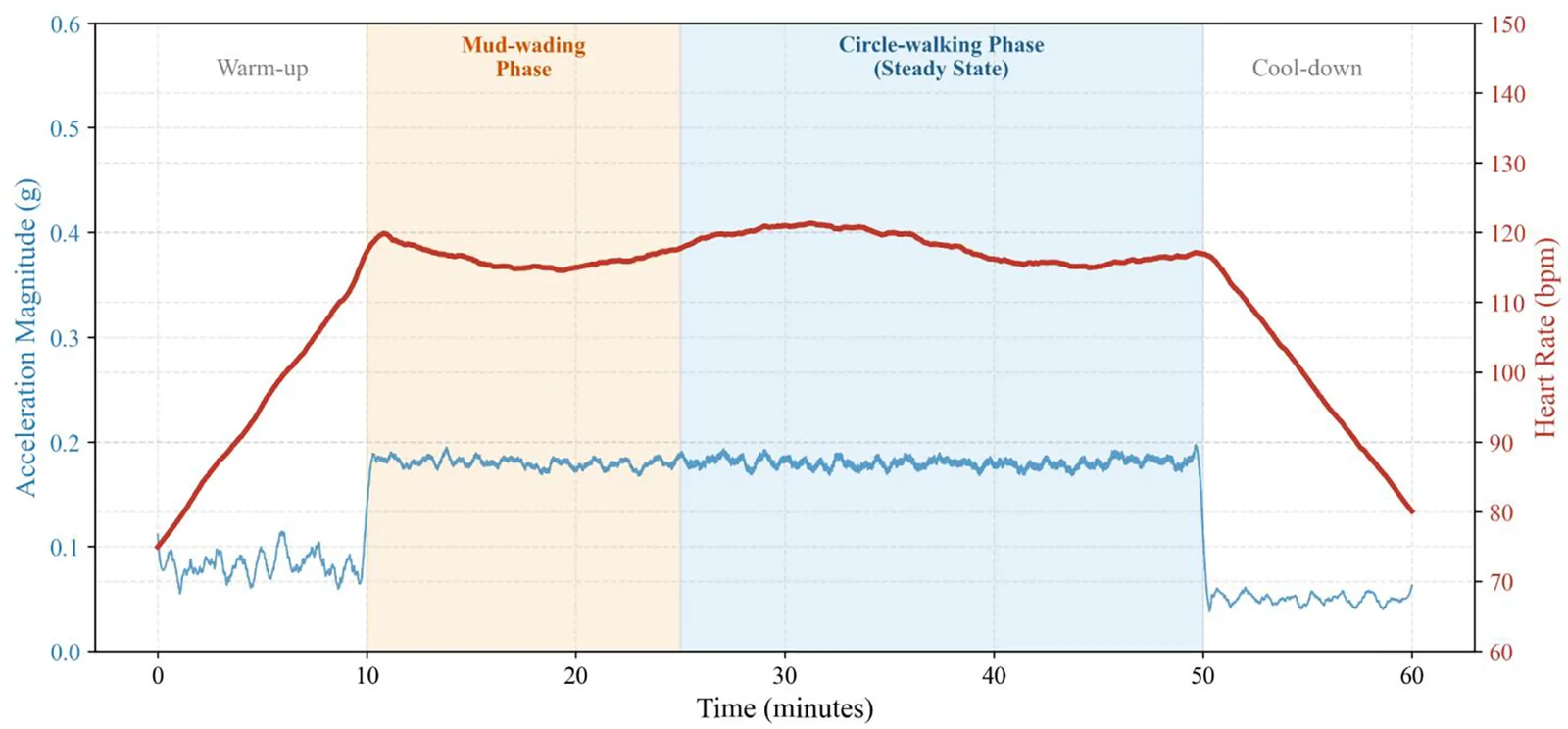
Representative synchronized heart-rate and waist-derived acceleration signals during a 60-min Baguazhang session. The session comprised a 10-min warm-up, a 15-min mud-wading block, a 25-min circle-walking block, and a 10-min cool-down. The plot is illustrative of a single recorded session and is not used to estimate group-level phase responses; participant-level phase summaries are reported in **Table 3**.

**Table 3:** Phase-specific physiological and kinematic characteristics of Baguazhang training based on participant-level averages (n = 23).

Metric	Warm-up (10 min)	Mud-wading (15 min)	Circle-walking (25 min)	Cool-down (10 min)	Overall session (60 min)
Average heart rate (beats/min)	98.2 ± 6.5	116.4 ± 7.8	133.4 ± 9.2	106.1 ± 7.1	118.5 ± 8.4
Heart-rate reserve (%)	23.4 ± 4.1	43.0 ± 5.5	61.5 ± 7.4	32.1 ± 4.8	45.7 ± 6.2
Phase TRIMP (AU)	4.1 ± 1.2	12.8 ± 3.1	37.5 ± 5.6	5.3 ± 1.4	59.7 ± 9.3
Mean amplitude deviation (g)	0.17 ± 0.03	0.18 ± 0.04	0.22 ± 0.05	0.09 ± 0.02	0.18 ± 0.04
Step count (steps/block)	600 ± 65	1,050 ± 90	1,950 ± 150	650 ± 75	4,250 ± 320

Values are mean ± SD across 23 participant-level averages. For each participant, phase-specific metrics were averaged across all attended sessions with valid sensor recordings; 812 valid sessions contributed repeated observations within participants in total. Sessions were not treated as independent observations. TRIMP, training impulse; MAD, mean amplitude deviation; HRR, heart-rate reserve; AU, arbitrary units.

### 3.2 Sensor-derived training load

The overall session profile is summarized in **Table 4**. Mean heart rate across the full 60-min session was 118.5 ± 8.4 beats/min, corresponding to 45.7 ± 6.2% HRR and falling within the moderate-intensity range for aerobic exercise (Garber et al., 2011). Mean session TRIMP was 59.7 ± 9.3 arbitrary units (95% CI 56.0 to 63.4), representing a moderate internal cardiovascular load on average. Mean waist-derived MAD was 0.18 ± 0.04 g, and participants accumulated 4,250 ± 320 steps per session.

**Table 4:** Overall physiological and kinematic characteristics of Baguazhang training (n = 23).

Metric	Mean ± SD	95% CI
Duration (min)	60.1 ± 1.5	59.5 to 60.7
Average heart rate (beats/min)	118.5 ± 8.4	115.1 to 121.9
Peak heart rate (beats/min)	142.3 ± 10.2	138.1 to 146.5
Heart-rate reserve (%)	45.7 ± 6.2	43.1 to 48.3
Session TRIMP (arbitrary units)	59.7 ± 9.3	56.0 to 63.4
Mean amplitude deviation (g)	0.18 ± 0.04	0.16 to 0.20
Step count (steps/session)	4,250 ± 320	4,119 to 4,381

CI, confidence interval; TRIMP, training impulse. Values are mean ± SD of participant-level session averages. Mean session TRIMP characterizes typical attended-session load and was not used as a reproducibility statistic.

Phase-specific analysis based on participant-level averages from the 812 valid sessions is presented in **Table 3**. The circle-walking block showed the highest mean cardiovascular demand among the four session blocks: heart rate was 133.4 ± 9.2 beats/min, intensity was 61.5 ± 7.4% HRR, and phase TRIMP was 37.5 ± 5.6 AU. Corresponding %HRR values were 23.4 ± 4.1% during warm-up, 43.0 ± 5.5% during mud-wading drills, and 32.1 ± 4.8% during cool-down. Thus, the circle-walking block contributed the largest share of session TRIMP and showed the highest phase-specific heart-rate response.

Waist-derived MAD was 0.17 ± 0.03 g during warm-up, 0.18 ± 0.04 g during mud-wading drills, 0.22 ± 0.05 g during the circle-walking block, and 0.09 ± 0.02 g during cool-down. These values describe acceleration amplitude at the waist and do not establish low vertical displacement, low ground-reaction force, low joint loading, or reduced musculoskeletal impact. The weekly intensity heat map in **Figure 6** provides a descriptive view of participant-level intensity across the intervention, and **Figure 3** shows weekly mean cumulative TRIMP and pre-session resting heart rate; the weekly load values appeared descriptively similar rather than being interpreted as a formal measure of reproducibility.

**Figure 6:**
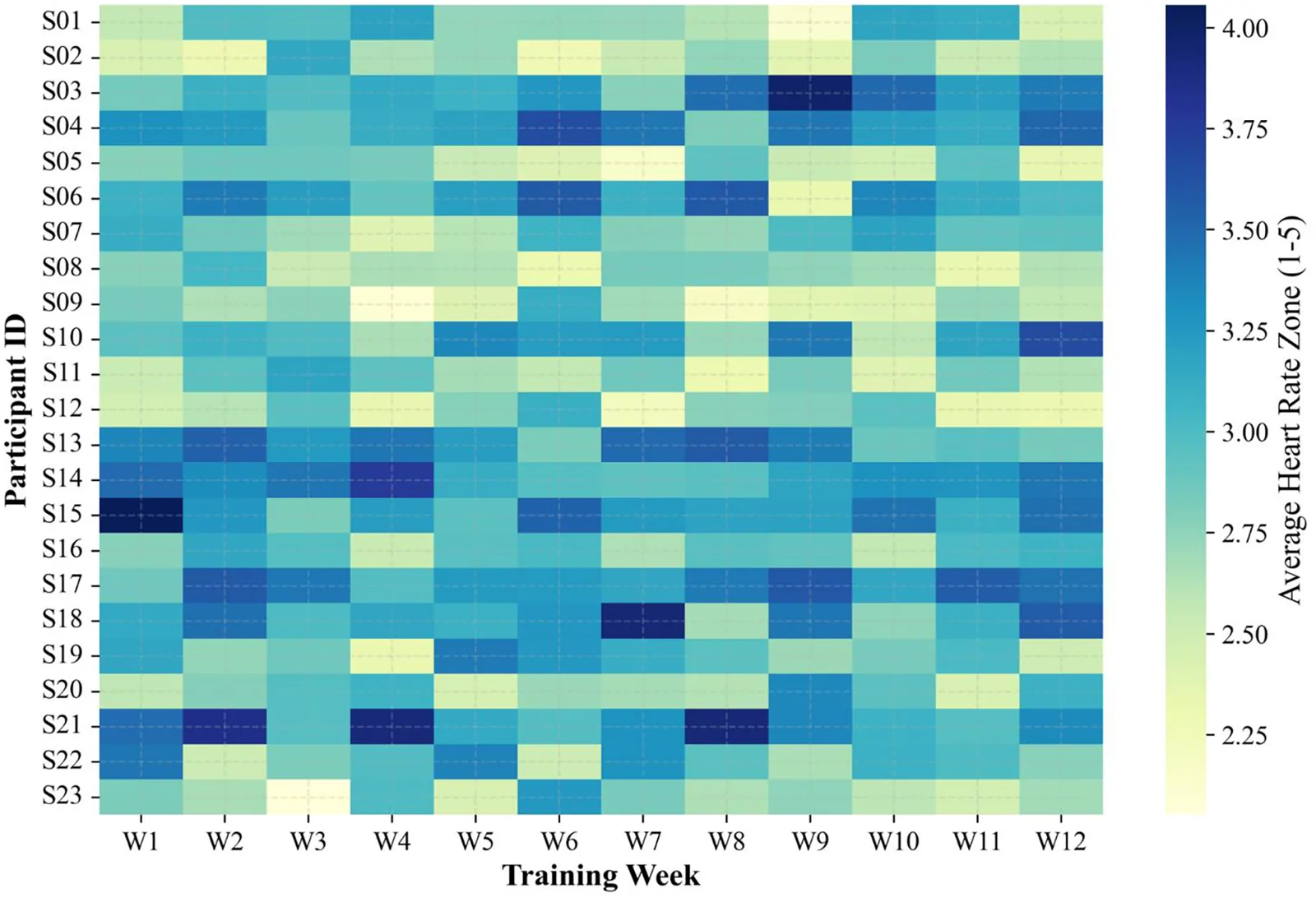
Participant-level distribution of average heart-rate zones across the 12-week Baguazhang intervention. Rows represent the 23 participants (S01-S23), columns represent training weeks (W1-W12), and color intensity represents the participant-specific average heart-rate zone on a five-zone scale derived from %HRR. The zone values are descriptive summaries of weekly intensity and were not analyzed as continuous outcomes.

### 3.3 Cardiovascular, autonomic, and body-composition outcomes

The primary and secondary outcome estimates are shown in **Table 5**. For the primary outcome, baseline-adjusted SBP was 7.2 mmHg lower in the Baguazhang group than in the habitual-lifestyle control group at 12 weeks (95% CI −10.5 to −3.9; p < 0.001). Supportive secondary analyses, for which p values were nominal and not adjusted for multiplicity, yielded adjusted between-group differences in DBP (−4.4 mmHg, 95% CI −7.2 to −1.6; p = 0.004), resting heart rate (−4.5 beats/min, 95% CI −6.8 to −2.2; p = 0.001), and LnRMSSD (+0.31, 95% CI 0.12 to 0.50; p = 0.002). Smaller nominal differences were observed for body mass index (−0.7 kg/m², p = 0.038) and body-fat percentage (−0.9 percentage points, p = 0.015). These secondary findings are interpreted as supportive rather than confirmatory. Individual participant data for SBP, DBP, and LnRMSSD are shown in **Figure 7**.

**Table 5:** Pre- and post-intervention health outcomes and ANCOVA-adjusted between-group effects.

Variable	Group (n=23/group)	Baseline mean ± SD	Post-intervention mean ± SD	Adjusted mean difference (95% CI)	p value	Partial η²
Body mass index (kg/m²) [secondary]	Baguazhang	24.8 ± 3.1	24.2 ± 2.9	–0.7 (–1.3 to –0.1)	0.038	0.10
	Control	25.1 ± 3.4	25.3 ± 3.3			
Body fat (%) [secondary]	Baguazhang	34.2 ± 4.5	33.4 ± 4.2	–0.9 (–1.6 to –0.2)	0.015	0.13
	Control	34.5 ± 4.8	34.7 ± 4.6			
Systolic blood pressure (primary outcome; mmHg)	Baguazhang	134.5 ± 9.2	127.7 ± 8.5	–7.2 (–10.5 to –3.9)	<0.001	0.25
	Control	133.8 ± 8.9	134.2 ± 9.1			
Diastolic blood pressure (mmHg) [secondary]	Baguazhang	82.3 ± 6.4	78.5 ± 5.8	–4.4 (–7.2 to –1.6)	0.004	0.18
	Control	81.9 ± 7.1	82.5 ± 6.8			
Resting heart rate (beats/min) [secondary]	Baguazhang	76.4 ± 5.5	72.2 ± 4.8	–4.5 (–6.8 to –2.2)	0.001	0.22
LnRMSSD [secondary]	Baguazhang	3.31 ± 0.22	3.51 ± 0.20	+0.31 (0.12 to 0.50)	0.002	0.20
	Control	3.34 ± 0.19	3.32 ± 0.21			

LnRMSSD, log-transformed root mean square of successive differences. Adjusted mean differences (Baguazhang minus Control) and p values are derived from ANCOVA models adjusting for baseline values. SBP was the primary outcome. All other outcomes were secondary; their p values are nominal and were not adjusted for multiple comparisons.

**Figure 7:**
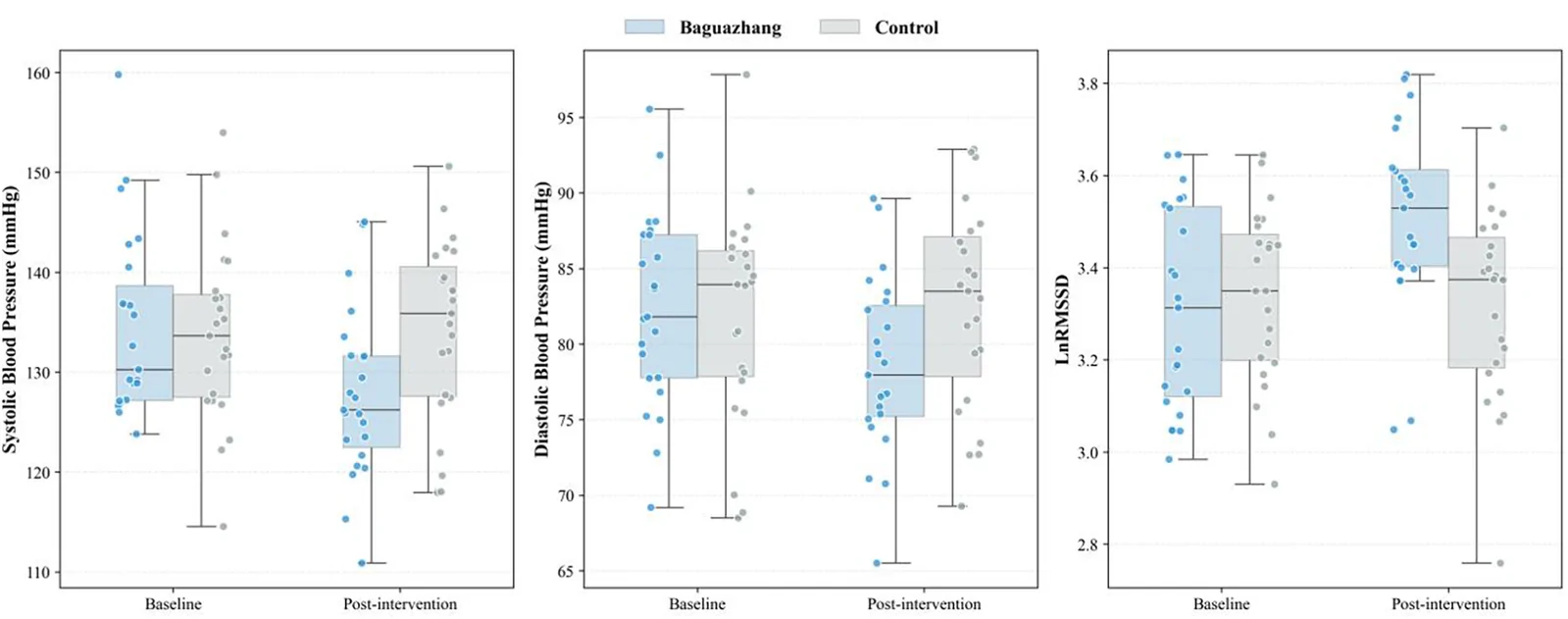
Individual participant data and summary statistics for cardiovascular and autonomic outcomes. Boxplots illustrate the median and interquartile range (IQR), while overlaid scatter points display the distribution of individual values for the Baguazhang group (blue) and control group (grey) at baseline and post-intervention. Left panel: systolic blood pressure; middle panel: diastolic blood pressure; right panel: log-transformed root mean square of successive differences (LnRMSSD).

### 3.4 Exploratory associations between cumulative load and physiological change

Exploratory participant-level associations are summarized in **Table 6** and **Figure 8**. Cumulative 12-week TRIMP was associated with a change in resting heart rate (r = −0.582, 95% CI −0.80 to −0.22, p < 0.01). Change in LnRMSSD was associated with a change in SBP (r = −0.512, 95% CI −0.77 to −0.13, p = 0.01), whereas mean MAD showed a weaker non-significant association with a change in body-fat percentage.

**Table 6:** Exploratory correlations between sensor-derived measures and physiological changes in the Baguazhang group (n = 23).

Variable pair	Pearson r (95% CI)	p value	Interpretation
Total TRIMP vs. change in resting heart rate	−0.582 (−0.80 to −0.22)	<0.01	Greater accumulated internal load was correlated with a larger reduction in resting heart rate.
Total TRIMP vs. change in systolic blood pressure	−0.420 (−0.71 to −0.01)	0.046	Greater accumulated internal load was correlated with a larger reduction in systolic blood pressure.
Mean MAD vs. change in body fat percentage	−0.310 (−0.64 to +0.12)	0.15	Mean MAD showed a weak, non-significant association with change in body fat percentage.
Change in LnRMSSD vs. change in systolic blood pressure	−0.512 (−0.77 to −0.13)	0.01	Change in LnRMSSD was correlated with change in systolic blood pressure; this bivariate association does not establish mediation.

Correlation p values are unadjusted for multiple comparisons. Negative values indicate that higher load or larger autonomic change was associated with a greater decrease in the paired outcome. These bivariate associations do not establish causality, dose-response, or mediation.

**Figure 8:**
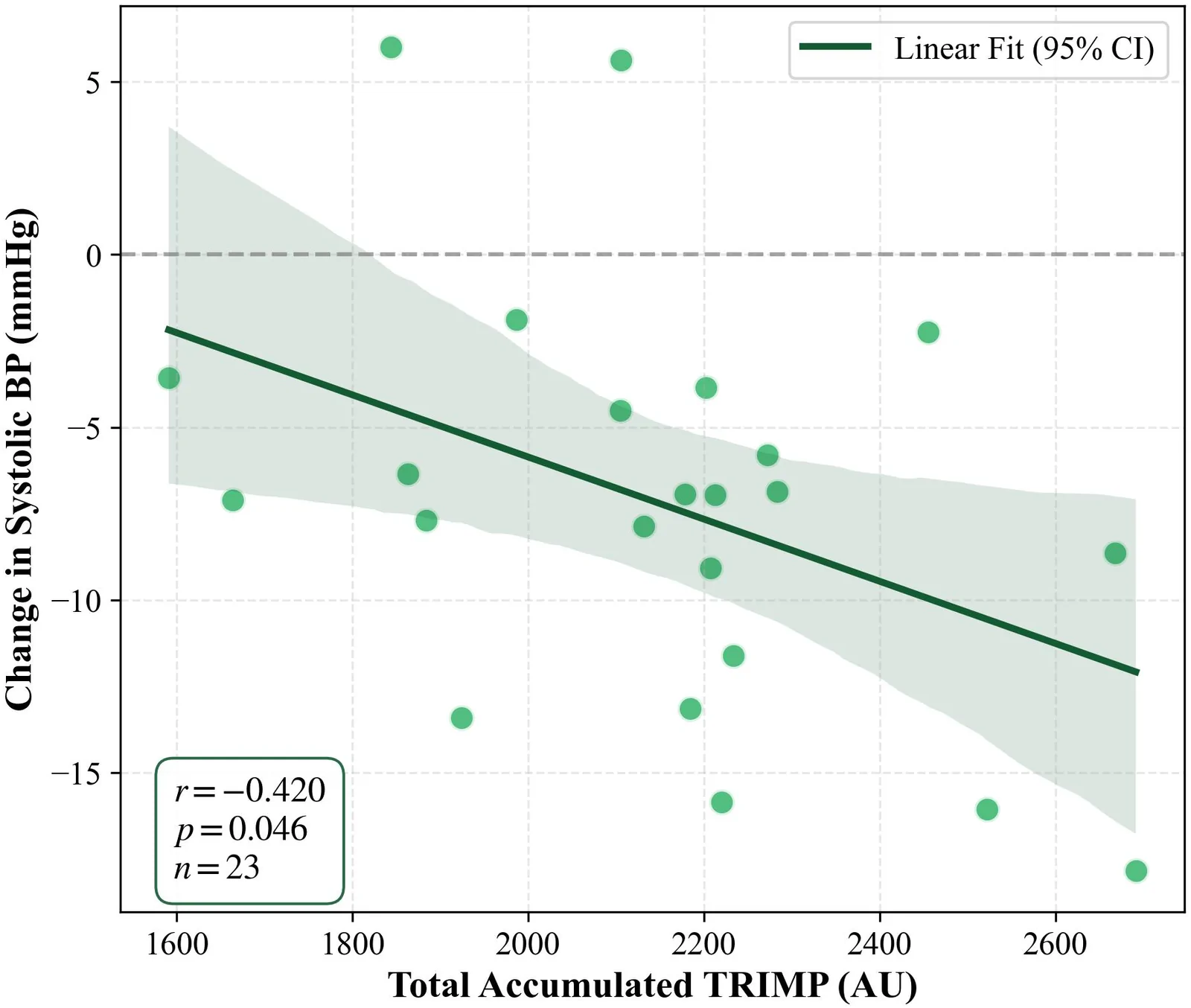
Exploratory association between cumulative 12-week training impulse and change in systolic blood pressure in the Baguazhang group (n = 23). Each point represents one participant. Negative change values indicate a reduction in systolic blood pressure from baseline to post-intervention. The solid line shows the linear fit and the shaded band its 95% confidence interval (Pearson r = −0.420, p = 0.046). The analysis was exploratory and was not adjusted for multiple comparisons.

To account for baseline values and regression to the mean regarding the primary outcome, a parsimonious multivariable linear regression was performed using post-intervention SBP as the dependent variable. The model included cumulative 12-week TRIMP and baseline SBP as predictors. The overall model explained a significant portion of the variance (Adjusted R² = 0.412, p = 0.002). Baseline SBP was a significant covariate (B = 0.58, 95% CI: 0.22 to 0.94, p = 0.003). After adjusting for baseline SBP, total accumulated TRIMP remained negatively associated with post-intervention SBP (B = −0.007, 95% CI: −0.013 to −0.001, p = 0.028). Regardless of significance, these exploratory analytical findings do not establish a causal dose-response relationship, nor do they prove mediation of the blood-pressure effect by autonomic change.

## 4 Discussion

This study combined synchronized wearable biosensors with a randomized 12-week intervention to characterize Baguazhang in sedentary postmenopausal women. The main findings were that the full session elicited moderate average cardiovascular intensity, the circle-walking block showed the greatest phase-specific cardiovascular demand, and the primary outcome of SBP was lower after Baguazhang than after habitual lifestyle. Supportive secondary analyses suggested favorable changes in DBP, resting heart rate, and LnRMSSD, but these outcomes were not adjusted for multiplicity. Waist accelerometry characterized the trunk-acceleration pattern of the practice and should not be interpreted as a direct measure of musculoskeletal impact.

### 4.1 Multicomponent movement structure and phase-dependent cardiovascular demand

Baguazhang combines circular locomotion, repeated directional changes, sustained lower-limb postural control, trunk rotation, coordinated palm changes, and breathing regulation within a single practice. This movement structure differs from steady straight-line walking at the level of task composition and is consistent with a multicomponent exercise format involving aerobic locomotion, postural control, dynamic balance, and motor coordination. However, the present study did not directly measure balance performance, lower-limb muscular endurance, motor learning, acceptability, or psychological outcomes, and it did not include an active exercise comparator. The data therefore characterize how Baguazhang is performed and loaded physiologically, but do not establish unique health benefits or superiority over walking or other moderate-intensity exercise.

The phase-specific analysis clarifies the physiological contribution of the session components. The circle-walking block produced the highest mean heart rate (133.4 beats/min), the highest mean %HRR (61.5%), and the largest phase TRIMP (37.5 AU), whereas the warm-up, mud-wading, and cool-down blocks showed lower cardiovascular demand. The overall 60-min session nevertheless averaged 45.7% HRR, supporting a moderate mean cardiovascular intensity. MAD ranged from 0.09 g during cool-down to 0.22 g during circle walking. These values describe waist-derived acceleration amplitude only; without a comparator activity or a validated threshold for musculoskeletal impact, they should not be classified as evidence of low ground-reaction force, low joint loading, or a low-impact mechanism.

### 4.2 Cardiovascular adaptations and clinical relevance

The 7.2-mmHg adjusted between-group reduction in SBP, the primary outcome, may be clinically meaningful in magnitude. Meta-analytic evidence indicates that blood-pressure lowering is associated with lower cardiovascular-event risk at the population level (Ettehad et al., 2016), but cardiovascular events and formal risk outcomes were not assessed in this trial. The present result therefore supports a favorable short-term SBP response to supervised Baguazhang compared with habitual lifestyle. Because the control group did not perform an active exercise program, the study does not determine whether the SBP response differs from that produced by walking or another moderate-intensity intervention.

While the vascular mechanisms underlying the observed SBP reduction were not directly measured in the present study, the blood-pressure response is broadly consistent with meta-analytic evidence showing that exercise training reduces blood pressure in postmenopausal women (Yang et al., 2024). Plausible explanations include exercise-related improvements in endothelial function, nitric oxide bioavailability, and arterial stiffness (Green et al., 2004). Because these vascular parameters were not assessed, these mechanisms remain plausible explanations rather than demonstrated mechanisms of Baguazhang. Similarly, the association between change in LnRMSSD and change in SBP does not demonstrate autonomic mediation of the blood-pressure response.

### 4.3 Autonomic regulation and mind-body characteristics

Supportive secondary analyses showed nominal between-group differences in resting heart rate, DBP, and LnRMSSD, but these outcomes were not adjusted for multiplicity. The higher LnRMSSD observed after the intervention represents a higher value of this time-domain HRV index, while the lower resting heart rate is consistent with a lower resting cardiac rate after training. LnRMSSD is not a direct measure of cardiac vagal or sympathetic neural activity. These findings should therefore be interpreted as supportive physiological signals rather than confirmatory evidence of autonomic or cardioprotective effects. The smaller nominal differences in body mass index and body-fat percentage likewise require caution because they were secondary outcomes and their p values were relatively close to the conventional 0.05 threshold.

The coordinated breathing, visual focus, trunk rotation, and continuous stepping inherent to Baguazhang provide a rationale for future work on autonomic regulation, motor control, and exercise acceptability. Prior systematic reviews and meta-analyses have directly examined heart-rate-variability responses to Tai Chi and related mind-body practices, including time-domain indices such as RMSSD (Zhou et al., 2024; Gunawan et al., 2026). These studies provide relevant context for the present LnRMSSD result but do not establish that the same autonomic mechanisms occurred during Baguazhang training. Breathing was not mechanically paced during the resting autonomic assessment, and LnRMSSD was an unadjusted secondary outcome. Accordingly, the observed LnRMSSD change should not be interpreted as direct evidence that Baguazhang enhanced cardiac vagal activity, reduced sympathetic activity, or mediated the reduction in SBP. Future studies incorporating paced breathing, baroreflex sensitivity testing, and formal mediation analyses are needed to investigate these possibilities.

### 4.4 Value of wearable sensor fusion for traditional movement practices

Combining electrocardiographic and inertial sensing provided complementary information about Baguazhang. Heart-rate-derived measures quantified internal cardiovascular strain, whereas waist accelerometry described the accompanying trunk-acceleration pattern. Neither signal alone fully characterizes this multicomponent movement practice, and the two measurement domains should remain conceptually distinct: TRIMP reflects internal cardiovascular load, whereas MAD reflects acceleration variability at the waist rather than tissue or joint loading.

This combined monitoring approach may be useful for verifying exercise intensity in supervised or community settings and for describing how different movement blocks contribute to session load. Future systems could add wrist and ankle inertial sensors, portable metabolic measurement, and validated balance or strength assessments to provide a more complete physiological and biomechanical characterization of Baguazhang.

### 4.5 Limitations and future directions

Several limitations should be considered. First, the habitual-lifestyle control group did not include an active exercise comparator. Consequently, the present study cannot determine whether the observed effects are specific to Baguazhang or are attributable to supervised moderate-intensity exercise in general. Therefore, the present findings do not imply that Baguazhang has unique benefits or is superior to other exercise modalities. Second, although Baguazhang has a multicomponent movement structure, balance, muscular endurance, motor coordination, exercise acceptability, and psychological outcomes were not directly measured. Third, secondary outcomes were not adjusted for multiple comparisons, and the participant-level correlation analyses were exploratory, unadjusted, and based on only 23 participants. Fourth, this study was not prospectively registered in a publicly accessible clinical trial registry. Although the protocol was approved and archived by the institutional ethics board (IRB approval code: 2025-BGZ-08) prior to any participant enrollment, the absence of prospective public registration remains a limitation regarding the independent public verification of our prespecified primary outcome (SBP) and statistical analysis plan. Fifth, diet, sodium intake, sleep, stress, and physical activity outside the intervention were monitored using self-reported logs and basic step counts rather than comprehensive objective monitoring. Sixth, the sample comprised relatively healthy sedentary postmenopausal women recruited from a single center, which limits generalizability. Seventh, waist-mounted MAD describes trunk acceleration but not vertical displacement, ground-reaction force, joint loading, loading rate, plantar pressure, or tissue mechanical stress. Eighth, the intervention lasted 12 weeks without long-term follow-up. Ninth, direct oxygen-consumption measurement was not included. Finally, Baguazhang-specific literature remains limited, so some contextual comparisons necessarily draw on related mind-body exercise research.

Future studies should use prospectively registered protocols, active exercise comparators, larger multi-center samples, and longer follow-up. Phase-specific cardiometabolic measurements, portable oxygen-consumption assessment, balance and muscular-endurance testing, and multi-segment inertial sensing would help determine whether the multicomponent structure of Baguazhang produces physiological or functional effects that differ from those of other moderate-intensity exercise modalities.

### 4.6 Clinical and practical implications

The present findings suggest potential applications for community health promotion, active-aging services, and fitness and wellness programs. For sedentary postmenopausal women similar to those enrolled in the present study, supervised Baguazhang may be considered as one option for moderate-intensity multicomponent physical activity. Its feasibility, safety, and effectiveness in other populations, including frail older adults and long-term-care residents, require specific evaluation before broader implementation.

Heart-rate- and TRIMP-based wearable measures may help practitioners quantify and monitor internal training load during supervised Baguazhang sessions. The present study did not establish validated TRIMP thresholds for exercise safety or clinical effectiveness; wearable monitoring should therefore be regarded as a tool for load quantification rather than as a means of ensuring a safe or effective cardiovascular stimulus. Practical implementation should include appropriate participant screening, professional supervision, progressive technical instruction to reduce potential injury risk, and ongoing safety monitoring. The feasibility, resource requirements, and cost-effectiveness of wearable monitoring were not evaluated and require further study.

These potential applications should be interpreted within the limits of the current evidence. The findings do not establish that Baguazhang is superior to other established exercise modalities, demonstrate unmeasured balance or musculoskeletal benefits, or support Baguazhang as a replacement for prescribed medical treatment.

## 5 Conclusion

Baguazhang elicited moderate average cardiovascular intensity across the 60-min session, with the highest phase-specific heart-rate response and largest TRIMP contribution during the circle-walking block. In sedentary postmenopausal women, 12 weeks of supervised Baguazhang training was associated with a lower SBP than habitual lifestyle. Supportive secondary outcomes suggested favorable changes in DBP, resting heart rate, and LnRMSSD but were not adjusted for multiplicity. Waist-derived MAD characterized trunk-acceleration amplitude and does not establish low joint or musculoskeletal loading. Together with 98.1% session adherence and the absence of reported adverse events, these findings support Baguazhang as a feasible, measurable, moderate-intensity multicomponent exercise option. They do not establish superiority over walking or other active exercise programs.

## Data Availability

De-identified participant-level outcome data and processed sensor-derived variables supporting the findings of this study are available from the corresponding author upon reasonable request. Raw continuous ECG/R-R and accelerometry recordings are not publicly deposited because these physiological time-series data may carry participant privacy and potential re-identification risks. Requests for de-identified data will be considered in accordance with applicable institutional ethics and data-protection requirements. Requests to access the datasets should be directed to JC, caoyinheng@outlook.com.
